# Pregnancy detection and monitoring in cattle via combined foetus electrocardiogram and phonocardiogram signal processing

**DOI:** 10.1186/1746-6148-8-164

**Published:** 2012-09-17

**Authors:** Gaetano D Gargiulo, Richard W Shephard, Jonathan Tapson, Alistair L McEwan, Paolo Bifulco, Mario Cesarelli, Craig Jin, Ahmed Al-Ani, Ning Wang, André van Schaik

**Affiliations:** 1 HEARD Systems, 502/143 York St., Sydney, NSW, 2000, Australia; 2 University of Western Sydney, Penrith, NSW, 2751, Australia; 3 The University of Sydney, Sydney, NSW, 2006, Australia; 4 “Federico II” The university of Naples, Naples, 80100, Italy; 5 University of Technology, Ultimo, Sydney, NSW, 2007, Australia; 6 University of New South Wales, Sydney, NSW, 2052, Australia

## Abstract

**Background:**

Pregnancy testing in cattle is commonly invasive requiring manual rectal palpation of the reproductive tract that presents risks to the operator and pregnancy. Alternative non-invasive tests have been developed but have not gained popularity due to poor specificity, sensitivity and the inconvenience of sample handling. Our aim is to present the pilot study and proof of concept of a new non invasive technique to sense the presence and age (limited to the closest trimester of pregnancy) of the foetus by recording the electrical and audio signals produced by the foetus heartbeat using an array of specialized sensors embedded in a stand alone handheld prototype device. The device was applied to the right flank (approximately at the intercept of a horizontal line drawn through the right mid femur region of the cow and a vertical line drawn anywhere between lumbar vertebrae 3 to 5) of more than 2000 cattle from 13 different farms, including pregnant and not pregnant, a diversity of breeds, and both dairy and beef herds. Pregnancy status response is given “on the spot” from an optimized machine learning algorithm running on the device within seconds after data collection.

**Results:**

Using combined electrical and audio foetal signals we detected pregnancy with a sensitivity of 87.6% and a specificity of 74.6% for all recorded data. Those values increase to 91% and 81% respectively by removing files with excessive noise (19%).

Foetus ageing was achieved by comparing the detected foetus heart-rate with published tables. However, given the challenging farm environment of a restless cow, correct foetus ageing was achieved for only 21% of the correctly diagnosed pregnant cows.

**Conclusions:**

In conclusion we have found that combining ECG and PCG measurements on the right flank of cattle provides a reliable and rapid method of pregnancy testing. The device has potential to be applied by unskilled operators. This will generate more efficient and productive management of farms. There is potential for the device to be applied to large endangered quadrupeds in captive breeding programs where early, safe and reliable pregnancy diagnosis can be imperative but currently difficult to achieve.

## Background

### Pregnancy testing of cattle

Pregnancy diagnosis is one of the most frequently performed diagnostic procedures undertaken on cattle [1,2]. Timely testing of individual cows for pregnancy supports optimal management of individual animals and the maximisation of farm profit for both the dairy and beef production systems [3,4]. The current recommendations from the major beef and dairy industry research and development organisations in Australia is for each mated female to be pregnancy tested at least once per year [3,4].

The two most frequently used methods for pregnancy diagnosis of cattle are manual palpation of the reproductive tract (per rectum) and transrectal ultrasonography of the reproductive tract [2]. Veterinarians and specialist animal technicians most commonly provide these services to farmers on a fee-per-cow or time charge basis. Both procedures are invasive and practitioners require extensive training in order to undertake the procedure safely (for cow and operator) at sufficient accuracy and speed for the service to be economically viable. An experienced practitioner using ultrasound can reliably diagnose pregnancy from 30 days gestation whilst an experienced manual palpater is able to diagnose pregnancy from 35 days. Both techniques can diagnose pregnancies from these points through to full term (282 days) with sensitivities and specificities exceeding 95% [5-7]. There is little advantage from detecting pregnancy before 35 days of gestation as the majority of embryonic loss – up to 1/3^rd^ of all conceptions – occurs from conception to day 35 [8]. Cows diagnosed as pregnant before day 35 of gestation should be re-examined again following the 35th day to ensure the pregnancy has been maintained. An experienced operator can provide foetus aging (to the week level) to 14 weeks of gestation for ultrasound and to 18 weeks of gestation for manual examination. Foetus aging beyond these stages of pregnancy is less accurate with most practitioners able to age the foetus to within the nearest month with acceptable accuracy; but only by manual examination [9].

Ideal conditions are often not present in the field and experienced practitioners are not always available for farmers (e.g. Northern Territory and South Australia remote farms). Test sensitivity (expressed as ratio between number of true positives and sum of the numbers of true positives and false negatives) and specificity (expressed as ratio between number of true negatives and sum of the numbers of true negatives and false positives) may be reduced due to the combined effects of operator skill level, operator fatigue, poor facilities and individual herd factors such as cow demeanour, cow body condition and diet. Both invasive methods may present risk to the pregnancy and to the cow, with abortion risk being greatest for manual palpation and for first trimester pregnancies [10]. Abortion following manual pregnancy diagnosis has been reported and one estimate of the attributable risk for abortion following manual pregnancy diagnosis for cows less than 42 days pregnant was 5% of pregnancies [11].

Alternative non-invasive and non-expert dependent methods of pregnancy diagnosis have been developed. These are generally assay-based tests (such as enzyme-linked immunosorbent assay (ELISA), radioimmunoassay (RIA) or latex agglutination (LA) tests). These tests use either blood or milk to detect a marker of pregnancy. Some of the markers that have been examined as indicators of pregnancy in cattle include oestrone sulfate [12,13], progesterone [14-16] and pregnancy associated glycoproteins (PAG) [17,18]. Oestrone sulfate is produced by the foetus and as such offer high specificity. However, these tests have not gained popularity due to the high rate of false negatives and the inability of the test to reliably diagnose pregnancies before 100 days of gestation [12].

Progesterone-based tests have also not been widely adopted. Progesterone is a naturally occurring hormone in non-pregnant as well as pregnant cows. The progesterone concentrations in serum and milk are related to ovarian luteal activity – being elevated during the luteal phase of the ovarian cycle but low (< 3 nmol/l) for approximately 4–5 days around the time of estrus. Estrus occurs approximately every 18–24 days in cycling and non-pregnant cows. Therefore the detection of low progesterone levels in samples obtained 18–24 days after insemination provides evidence that the corpus luteum has regressed and that a pregnancy has not occurred. Progesterone-based testing is therefore a secondary indicator of pregnancy with diagnosis requiring interpretation of carefully timed or serial samples obtained from individuals. This combined with the low sensitivity of the test has not resulted in widespread adoption [14].

Pregnancy associated glycoproteins are produced by the placenta and trophoblast and as such are direct indicators of pregnancy. These molecules appear in the circulation of pregnant cows from around 15 days after conception [17]. Levels of PAG can persist for some time after parturition and can result in false positive diagnoses in cows mated within eight weeks of calving. Up to 40% of all conceptions fail to be carried to term with the majority of these losses occurring at the early embryonic stage (< 35 days). The early rise of and persistence of levels of PAG in circulation of cows that have experienced early embryonic loss can also result in false positive diagnoses if cows are tested within seven days of experiencing embryonic loss. Other factors such as placental health, infection and the metabolic rate of the cow (related to her production level) appear to influence the levels of PAG in serum. Both the sensitivity and specificity of modern PAG-based tests can exceed 95% when used strategically [7].

Both manual and assay-based tests in their current forms have limitations to practical use. The necessity to use highly skilled contractors to undertake either manual or ultrasound-based pregnancy testing effectively limits usage. Most testing using these methods is applied at the herd level – little testing of individual animals is performed and generally this cannot be conducted on-demand. As a result, many animals are not tested at the optimal time for accurate diagnosis or for tailored management decisions to be made. In many countries and regions of the world (e.g. Australia) there is also a shortage of contractors to provide these services [19]. Rectal palpation is strenuous physical work for the operator. Increasing herd sizes increases the risk of fatigue-related and repetitive strain injuries amongst veterinarians and contractors providing whole-herd pregnancy testing services [20,21]. The high cost of ultrasound equipment and extensive training required to achieve a suitable level of competence is a barrier for entry for new contractors. In many countries and jurisdictions invasive pregnancy testing remains an act of veterinary science and non-veterinarians are unable to operate.

Assay-based tests are also problematic. Sample collection is invasive; sample collection may be restricted to trained professionals by legislation and often requires significant operator skill. Milk-based tests are also only suitable for lactating dairy cows and most current tests require samples to be sent for analysis to a laboratory thereby preventing an immediate diagnosis [22]. The advantage of cow-side testing (with immediate and real-time results) is that animals need only be handled once. Animals can be treated or drafted into management groups according to the results of the pregnancy test.

An ideal pregnancy diagnostic test for cattle would have the following features: high accuracy (sensitive and specific), non-invasive, easy and fast to administer, results available in real-time, safe for the operator and cow, no adverse effects on the pregnancy and most importantly able to be conducted directly by farmers and herd managers on demand. Other (optional) advantages include automatic digital data capture (that is linked with electronic animal identification system).

### Physical detection of the foetus heart beat

A system based upon the non-invasive detection of the foetus heart beat may allow the development of a real-time, non-invasive and rapid pregnancy diagnostic system for cattle. The foetus heart develops early in embryogenesis and displays regular beating by day 30 in cattle. The depolarisation of cardiac muscle tissue results in the dissemination of an electrical signal from the foetus through the maternal tissues. The activity of the heart and movement of fluid within blood vessels generates pressure and sound wave signals, which also disseminate from the foetus through the maternal tissues. The development of systems to capture, filter, process and analyze these various signal sources from detectors placed on the surface of the cow may allow the development of a general, non-invasive and farmer-operated pregnancy testing device for cattle.

The major problem of this approach is that any system must be able to detect these signals in the field. It is well known that detecting these foetus bio-signals in humans (e.g. electrocardiogram or ECG) outside a laboratory environment poses a unique set of challenges due to the weakness of the signals and the nonlinear and commonly unstable interface between the skin and the electrode connected to the electronic device [23,24].

Detecting heart sounds (phonocardiogram or PCG) also present extra challenges, the audio sensor must couple effectively to the body surface in order to capture and transmit the weak sound signal that is impinging upon the high acoustical impedance interface at the skin/sensor surface [25].

In this paper we present the detailed proof of concept study that led to the development of a farmer operated not invasive pregnancy detection device. Firstly we show that the foetal cardiac signals (ECG) are detectable as separate entity from the maternal ECG (ie. Time incoherence), then we show the advantage of combining ECG and PCG for pregnancy detection with an extensive data collection (more than 2000 cows including a variety of breeds spread over 13 testing farms).

## Results

### Study 1: Feasibility of foetus and maternal signal separation

**Figure 1:**
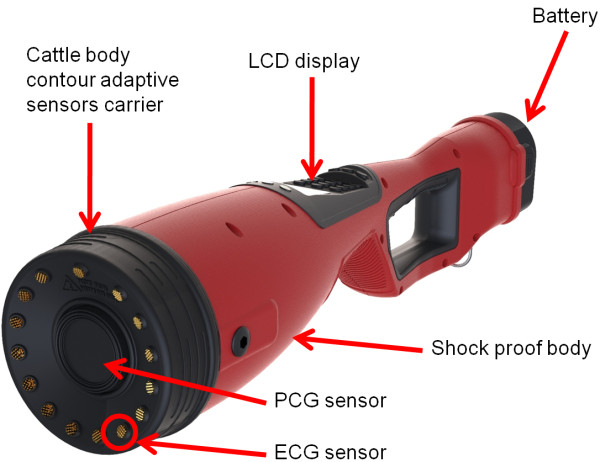
Prototype device unit and its parts.

**Figure 2:**
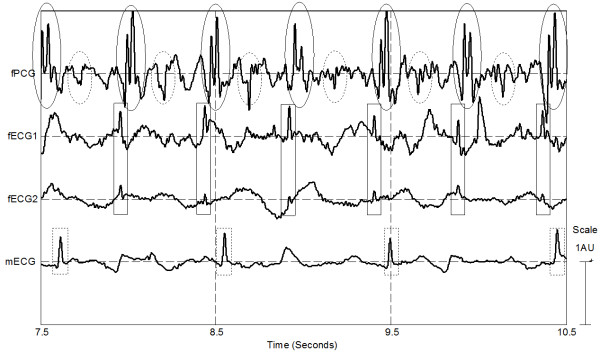
**Simultaneous recording of foetus PCG, foetus ECG and maternal ECG;** Top trace depicts foetus PCG; the solid circle highlights 1st (loud) cardiac sound and the dashed circle highlights the 2nd (weak) cardiac sound. The second and third traces from the top present the foetus ECG; the solid squares highlight the foetus QRS complexes. Maternal ECG (with QRS highlighted by dashed rectangles) is presented in the lower trace.

**Figure 3:**
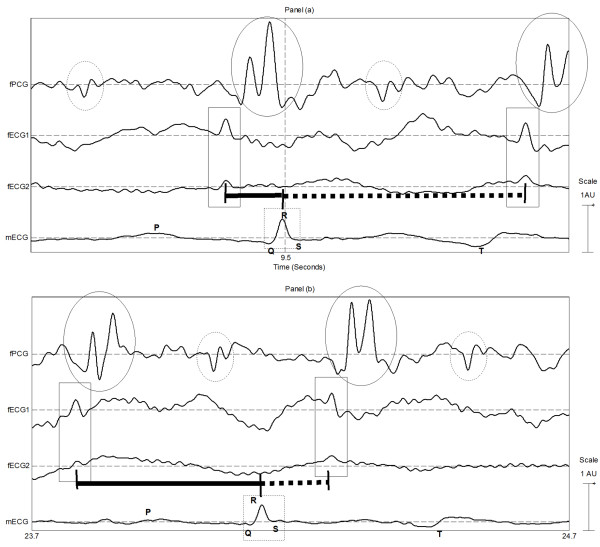
**Evidence of independence between maternal and foetus QRS events; Panel a: example of foetus QRS occurring just before the maternal QRS; Panel b: example of foetus QRS occurring just after the maternal QRS.** The top trace in both panels depicts foetus PCG; the solid circle highlights the 1st (loud) cardiac sound and the dashed circle highlights the 2nd (weak) cardiac sound. The second and third traces from the top present the foetus ECG; solid squares highlight the foetus QRS complexes. Maternal ECG (QRS highlighted with dashed rectangles) is depicted in the bottom trace.

The results for our pilot study are reported in Table 1 (the device used for this study is represented in Figure 1). Examples of combined recording of foetus signals (PCG and ECG) and maternal ECG from a 16-week pregnant cow are depicted in Figure 2 and Figure 3.

**Table 1:** Study 1 result

**Cow ID (HEARD System records)**	**Pregnant [P]; Not Pregnant [NP] manual palpation / farm record**	**Foetus age (farm record confirmed by palpation)**	**Average (1 minute) maternal HR [bpm]**	**Average (1 minute) foetus HR [bpm]**	**fHR/mHR**
Sub_01_1209	P	25 weeks	70	156	2.23
Sub_02_1209	P	22 weeks	66	155	2.36
Sub_03_1209	P	24 weeks	78	148	1.89
Sub_04_1209	P	28 weeks	56	143	2.55
Sub_05_1209	P	6 weeks	75	168 (PPG not detectable)	2.25
Sub_06_1209	P	26 weeks	62	143	2.30
Sub_01_0508	P	20 weeks	71	150	2.10
**Sub_02_0508**	**P**	**16 weeks**	**64**	**122**	**1.90**
Sub_03_0508	P	32 weeks	68	110	1.62
Sub_01_0909	P	12 weeks	81	158 (PPG not detectable)	1.95

Highlighted in bold are the data used to plot the example Figures 2 and 3.

### Study 2: Advantage in combining ECG and PCG signals for pregnancy detection

Evidence of the advantage of combining the recording of foetus ECG with foetus PCG is summarized in Table 2.

**Table 2:** Performance comparison between combined and separate electrical (ECG) and audio (PCG) foetus sensing for pregnancy detection

**Recording**	**Sensitivity**	**Specificity**
ECG only (no rejections)	85.2%	70.1%
PCG only ≥ 20 weeks	87.7%	73.2%
ECG and PCG combined (19% rejected)	87.6%	74.6%

As it is possible to infer from Table 2, the over-all performance of the prototype device tested on more than 2000 cattle (data sample is been collected from several different farms and includes beef and dairy heards) is greater than 80%. Device performances when only the ECG is been used are more sensitive than the performances evaluated using the PCG alone. The reason is that the PCG is reliably detected for foetus age ≥ 20 weeks. Therefore, the increase in performances in combined classification is greater for the specificity which increases of 4.1% while the sensitivity only increases of 2%.

## Discussion

### Development of a suitable sensor

A device was developed that can capture and process combined ECG and PCG signals obtained from a combination sensor array placed against the flank of the cow.

The device is battery powered, is completely passive (it only senses signals and does not inject a signal into the body of the cow), and it consists of two parts. The most important part of the device is the sensor head, which is designed to keep metallic electrode sensors supported by a rubber boot in contact with the cow. The sensor head is then connected with a device body, which offers a wide handle grip for the operator and contains all the remaining electronic parts and battery of the device. The device weighs approximately 3.5 kg (including battery) and measures approximately 15 cm at the largest diameter and approximately 60 cm in length.

Previous foetus ECG studies have been successful at monitoring pregnancies in the second and third trimesters in animals [26]. We have found that pregnancy may be detected using solely ECG or PCG [27] but that by combining these modalities we improve the sensitivity, specificity and reduce the time required for pregnancy detection. The device we have developed is held by the operator and applied to the flank of the animal with sufficient pressure to ensure adequate contact by both ECG and PCG sensors. Signals are processed by an on-board novel analogue to digital circuit and sent for further processing by a specific detection algorithm program operating on an on-board central processor.

### ECG

An ECG lead comprises two electrodes applied to the skin of the subject along with its amplification system [23,25]. The physical characteristics of the electrode are vital as this is the interface between the body and the electronic device and the quality of the contact determines (in part) the quality of the signal. There are two general classes of electrodes: ‘wet’ electrodes, and ‘dry’ electrodes.

Wet electrodes were the first to successfully detect ECG signals from the body. These rely on the electrical conductivity mechanism used in the body, where ions act as charge carriers. In order to detect bioelectric signals it is necessary to interact with these ionic charge carriers, and then transduce ionic currents into electric currents that can travel along wires and be processed by electronic instrumentation. The process is facilitated when the ionic charge is transduced into a current in a galvanic cell, formed by the skin on one side and a metal electrode on the other [28]. Unfortunately, in order to facilitate a stable reduction-oxidation (red-ox) reaction at the electrode level, the electrode is usually applied with a wet conductive gel or paste. These operate most effectively when surface hair is removed – effectively making this option impractical for a herd-level animal pregnancy detection device [27].

A dry electrode is one where no paste or conductive gel is required to provide effective contact with the skin. Effectively avoiding the need for conductive pastes and gels offers clear advantages including: no need for skin preparation, no gel desiccation, no electrolyte smearing resulting in electrical shorting between electrodes. However, the use of dry electrodes presents its own set of problems. For example, effective contact between the electrode and the skin is more variable when applied to unprepared skin. If the surface of the skin is irregular, a flat dry electrode may only have small points of contact with the skin [29]. This results in a smaller and less effective contact area than desired [30].

To compound the problems described, the recording of foetus ECG from sensors placed on the maternal skin is more challenging because the amplitude of the foetus ECG signal is10-100 times lower than the amplitude of the maternal ECG. The foetus ECG amplitude is generally less than 50 μV. We have developed a novel device based on the combination of an innovative paste-less dry electrode and suitable bio-potential amplifier to manage the issues described above.

### PCG

The heart sound signal or phonocardiogram (PCG) is perhaps the most traditional biomedical signal, as indicated by the prominence of the stethoscope as a diagnostic instrument used by medical and veterinary physicians. The normal heart sounds (in adults) may provide an indication of the general state of the heart in terms of rhythm and contractility [28,31,32].

The PCG is a vibration (sound) evoked by the contractile activity of the heart and the resultant hydrodynamic movement (blood flow). Recording of a PCG signal requires a transducer to convert the vibration or sound signal into an electronic signal: microphones, pressure transducers, or accelerometers may be placed on the body surface for this purpose.

The PCG signal has a characteristic shape and temporal relationship to the concurrent ECG signal. The combined capture and analysis of these signals from the body surface of the cow may be used to gather information about the pregnancy status of the cow. Any normal cardiac cycle (including the foetus heart) produces two major sounds. The first heart sound occurs at the onset of ventricular contraction, and corresponds in timing to the QRS complex in the ECG signal. Following the systolic pause (clearly visible in the PCG signal), the second sound is caused by the sequential closure of the aortic and pulmonary valves [33].

### Discussion on the data

Time domain separation of foetus and maternal cardiac activities is indicated by the fHR/mHR ratios in Table 1 which are greater than unity and non-integers ruling out the possibility that these rates are harmonically related.

The excerpts from an example recording shown in Figures 2 and 3 indicate high synchrony between the two foetus signals (fECG and fPCG) and low correlation between these and the maternal (mECG) signal. In Figure 2 and Figure 3a tight time relationship of approximately 30 ms can be observed between foetus QRSs and the first foetus loud cardiac sound. Each panel of Figure 2 depicts approximately one complete maternal heart beat cycle. The two panels were taken from the same recording sessions and were recorded a few seconds apart. The solid line segment represents the time interval between the maternal QRS and the immediately preceding foetus beat. The dashed line segment represents the time interval between the maternal beat and the next foetus beat. The x-axis scale in the two panels is equal and so it can be seen by visual inspection that the time delays are not equal in the two panels of Figure 3. This further suggests that there is a low time domain correlation between the fECG and mECG signals.

Table 2 shows that the over-all performance of the prototype device tested on more than 2052 cattle from 13 different farms, including beef and dairy heards is greater than 80%. ECG is more sensitive PCG alone as PCG is only reliably detected for foetus ages ≥ 20 weeks. However it does lead to an overall increase in performances with combined ECG and PCG classification where the specificity increases by 10% and sensitivity increases by 1%.

The cows examined covered the ‘testable’ pregnancy range (including non-pregnant, and from 6-weeks to 9-months pregnant). This has confirmed that the methodology used is applicable in principle. However, in order to make the method robust and applicable for commercial field use, the specialised testing and processing device is undergoing refinement and improvement.

Specialised electronic devices, acoustical sensor and electrodes (patent pending) have been developed to allow effective recording (through the hair coat of unprepared live cow hide) in a portable and hand-held device. This device incorporates specialised software that operates to provide the operator with information on captured signal quality and to provide a pregnancy diagnosis in real-time.

**Figure 4:**
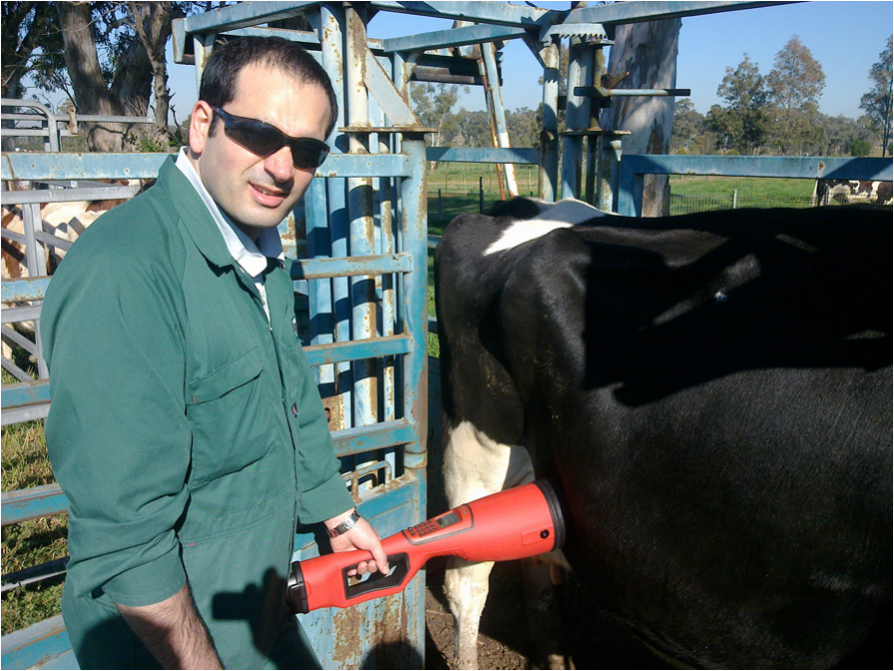
Example of use of prototype device (Courtesy of HEARD systems; the depicted subject kindly agreed to have his image published).

The device prototype (patent pending) has been successfully used across a number of field trials involving dairy and beef cattle, and the typical range of cattle handling facilities encountered on commercial dairy and beef farms. An example of how the device is applied to the flank of a cow is depicted in Figure 4. It can be seen that the operator is able to test cows that have been appropriately restrained in standard cattle crushes or chutes with safety for both operator and subject. The data collection procedure provides minimal stress to the animal, and being non-invasive provides minimal risk to the animal. These features combine to allow effective and safe testing of cattle by lay operators such as farmers.

The current version of the device uses a fixed data acquisition time of one minute per cow to ensure standardised data for algorithm development and refinement. Future versions will operate in real time and it is expected that a single operator will be able to diagnose approximately sixty cows per hour. It is expected that pregnancy testing may occur in parallel with other normal animal handling procedures, such as drenching and fertility examinations.

**Figure 5:**
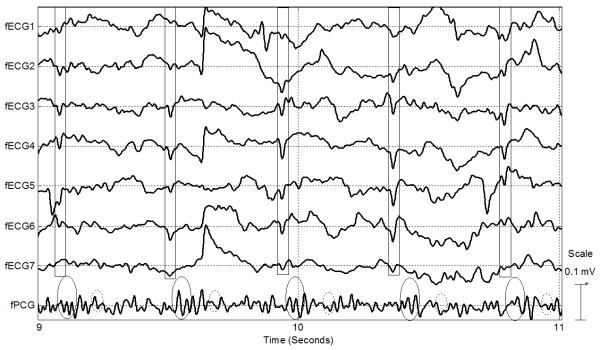
**Example of data acquired from a 20-week pregnant cow using the prototype device.** This device has eight channels (7 ECG and 1 PCG). The detected foetus QRS complexes are highlighted with solid squares across the ECG channels; first and second cardiac sounds are highlighted with a solid circles and dashed circles on the PCG channel respectively.

An excerpt of processed data acquired from the device is depicted in Figure 5. In the figure it is possible to clearly see six foetus heart beat events. Foetus ECG is especially evident in the channels “fECG5” and “fECG3” (represented in bold to facilitate the identification). The foetus ECG and PCG signal amplitude is variously a function of signal source size (i.e. foetus age), projection, coupling and electronic gain settings. The background noise (including maternal ECG/PCG, movement-generated artefacts and external noises such as 50 Hz mains power) is concurrently captured. For this reason sections of raw (and processed) traces contain variable foetus signal amplitudes. It is possible to recognise the foetus PCG pattern for the last three beats in Figure 5 – the foetus PCG signal is not visible beforehand due to the reasons described above.

An accurate diagnosis of pregnancy can be provided from recordings obtained from a single channel (either ECG or PCG) across five to ten seconds of good quality recording. Therefore there is much built in redundancy in the device (seven ECG channels and one PCG channel) and in the use pattern (one minute recording duration). This ensures that for the majority of recordings at least one diagnostic quality recording time window is obtained.

ECG leads redundancy it is required also because of the random orientation of the foetus inside the uterus. This is consistent with the basic principles of electrocardiography where different leads record different shapes of the ECG trace because of the different projections of the heart’s equivalent dipole as observable from the specific lead [23,24]. This principle accounts for the different shape and amplitude of the foetus ECG as seen in Figure 2 (labelled “fECG1” and “fECG2”).

Detecting foetus PCG is, to some extent, more difficult than detecting foetus ECG using this device. We found that the foetus PCG signals could be detected reliably only in heavily pregnant cows (gestation stage ≥ 20 weeks). During the first and part of the second trimester of pregnancy the foetus heart appears to be too small to disseminate a strong and reliable sound pressure wave that can be reliably detected using external sensors. It seems that the second cardiac sound is typically not detectable in early stage pregnancies. The classification software will therefore require further development to detect these weaker and incomplete PCG signals captured from early stage pregnancies.

It is important to note that the prototype device contained only one audio sensor and seven ECG leads (see Figure 5). Hence PCG signal detection and signal quality are totally dependent upon this single sensor’s performance and the quality of sensor coupling with the cow’s body.

The PCG signal is particularly sensitive to coupling quality, cow movement and other noise (e.g. subject vocalization, respiratory and gut sounds, etc.) and environmental noises (e.g. machinery, operator speech). These factors resulted in the rejection of a large number of PCG signal sections.

The concurrent manual palpation pregnancy test conducted by an experienced veterinarian was used as the gold standard comparator. The desired target sensitivity and specificity for a commercial pregnancy test in cattle is 95% or greater for both. The relatively lower sensitivity and specificity obtained in this study most likely arise from an excess of noise in the obtained signals. The most common source of noise identified was poor sensor contact (ECG and PGC) with the cow, cow movement artefacts (generating EMGs) and other physiological noise artefacts (EGG, skin twitching, vocalisation, etc.).

When excessively noisy data was removed from analysis (approximately 19% of recordings), the sensitivity and specificity increased to 89.4% and 91.5% respectively. The current priorities are: to refine the hardware system to capture more reliable signal with a higher signal-to-noise ratio (SNR); to improve the operator feedback software such that more precise feedback is provided to the user whilst operating the device (thereby guiding better placement of the device against the cow in real time); and to improve the performance of the decision software algorithm. These refinements and developments are on-going and a continual process of improvement and field testing is occurring to optimise the device.

## Conclusions

A novel, farmer-operated, hand-held and non-invasive pregnancy diagnosis system based on combined foetus ECG and PCG signal recording, and the associated proof of concept study is presented. This work has led to the development of a prototype hardware and software system incorporated into a portable, hand-held, stand-alone pregnancy test device for cattle that is suitable for use by a non-skilled operator in commercial herds.

The current device performance across a representative dataset obtained from commercial beef and dairy herds in Australia provided a global sensitivity and specificity of 83.9% and 79.1% respectively. Performance increased when poor quality data recordings were removed (approximately 35% of the current data set) to a sensitivity of 89.4% and a specificity of 91.5%.

Improvements in hardware (especially sensors) and software (operator feedback and detection algorithms) are expected to increase the proportion of captured signals that can be effectively processed. The objective is to achieve sensitivity and specificity in excess of 95% across the range of pregnancies, cattle and facilities found on commercial farms whilst operating at speeds of up to 60 cows per hour. Significant gains in signal quality are currently being achieved through the combined advancements in hardware and software.

## Methods

In this study we used the hand-held pregnancy detection device introduced above with custom manufactured audio and electrical sensors arranged in a circular array (patent pending) to perform two studies. In the first study we used two synchronized device to prove separation of maternal and foetus cardiac signals; in the second study we used only one device to simultaneously record foetus ECG and PGC on a large number of cattle and calculated the device performances as test sensitivity and specificity. The device (depicted in Figure 1) is totally battery powered and composed by a shock-proof body, a LCD display equipped with a mobile phone like keypad for the user interface, and a sensor array carrier. The sensor array is specifically designed to follow and adapt itself to the animal contour requiring only a moderate firm pressure to hold the sensors in sufficient contact. The array was specifically designed following pilot sensor mapping studies and the optimal location was found to be on the right flank approximately at the intercept of a horizontal line drawn through the right mid femur region and a vertical line drawn anywhere between lumbar vertebrae 3 to 5 (this position is hereafter referred as ‘foetus position’). The device was equipped with visual user feedback which constantly gave the user information about the data quality (sensor contact) and the length of the data recorded. This information was presented to the user on the device LCD screen. A loud buzzer signals that the device requires attention so that the user need not continuously observe the screen. Other useful information is always present on the LCD screen e.g. the last cow ID recorded, the usage count and the battery gauge.

The exposed electrical sensors on the sensor array were briefly submerged in water following each recording to remove dirt, hair and other debris.

### Study 1: Feasibility of foetus and maternal signal separation

We recorded both maternal and foetus PCG and ECG signals using two separate and synchronised prototype devices on ten pregnant subjects (as early as six weeks and up eight months pregnant) and ten not pregnant subjects. Foetal age was obtained from the farm management (insemination dates) and confirmed from expert practitioner (rectum palpation) on site after the recording. One device was placed on the foetus position and the second device was placed on the chest wall directly behind the right (fore) elbow of the cow as close as possible to the maternal heart (maternal position).

Averaged heart rates over 1 minute from each position were calculated as maternal and foetus heart rates (mHR and fHR) and the ratio fHR/mHR to test the separation of maternal and foetus signals.

### Study 2: Advantage in combining ECG and PCG signals for pregnancy detection

2052 cattle from 13 different farms were tested using a single device in the foetus position. This subject group included pregnant and not pregnant, a diversity of breeds, and both dairy and beef herds. The device was placed for a duration of one minute whilst the cow was suitably restrained in a crush/shute.

Then the diagnosis obtained by the device was compared to the true pregnancy status of each cow. The true pregnancy status was defined as a combination of herd mating records and manual pregnancy tests by an experienced veterinarian. This was considered to be the best gold standard that could be obtained, with the caveat that these methods (as mentioned) are not themselves 100% reliable [1,2].

The device determined pregnancy using signal processing and machine learning algorithms. In summary these include removal of saturated portions of the signal (e.g. due to poor electrode contact), filtering of unwanted frequency bands, and extracting a set of trained features for an extreme learning machine (ELM) based two class classifier where the two classes are pregnant or not pregnant. Classification is performed on each five-second window with an overlap of 2.5 seconds. The overall confidence of the classifier performance for a given file is obtained by averaging the “probability-like” output of the classifier of all considered windows.

Note: Farmers and owners of the testing farms that agreed to participate in testing also agreed in that the data be used for scientific purposes.

## Competing interests

Gaetano D. Gargiulo and Richard W. Shephard are currently employed by HEARD Systems

Craig Jin and André van Schaik are HEARD Systems co-founders.

Jonathan Tapson, Alistair McEwan, Ning Wang and Ahmed Al-Ani worked as contractors to HEARD Systems.

## Authors’ contributions

All listed authors reviewed and contributed to this paper, specifically: GDG designed the hardware to collect the data, assisted with data collection, conducted preliminary data analysis and designed the proof of concept study. RWS designed the HEARD System approach and methods, designed and led data collection (including gold-standard manual pregnancy testing of some subjects). JT contributed to the hardware development and led data analysis. AME contributed to the hardware review and to data analysis. PB and MC contributed to the data analysis. CJ reviewed hardware, assisted with data collection and had a lead role in data analysis and algorithm development. AA-A and NW contributed to data analysis and development of decision algorithm. AvS contributed to the data collection and the production of the decision algorithm. All authors read and approved the final manuscript.
